# Engineered Exosomes-Mediated Transfer of hsa-miR-320a Overcomes Chemoresistance in Cervical Cancer Cells *via* Targeting MCL1

**DOI:** 10.3389/fphar.2022.883445

**Published:** 2022-04-04

**Authors:** Jinling Zhou, Yuanhe Wang, Lizhu Zhang, Qin Chen, Xiaojun Zhu, Peiyue Jiang, Nan Jiang, Wei Zhao, Baohua Li

**Affiliations:** ^1^ Department of Obstetrics and Gynecology, Women’s Hospital, Zhejiang University School of Medicine, Hangzhou, China; ^2^ Institute of Nanjing Nanxin Pharmaceutical Technology Research, Nanjing, China; ^3^ Department of Pathology, Women’s Hospital, Zhejiang University School of Medicine, Hangzhou, China; ^4^ Department of Clinical Laboratory, Women’s Hospital, Zhejiang University School of Medicine, Hangzhou, China; ^5^ Department of Biomedical Sciences and Tung Biomedical Sciences Centre, City University of Hong Kong, Hong Kong, Hong Kong SAR, China

**Keywords:** engineered exosomes, cervical cancer, has-miR-320a, Mcl1, chemoresistance

## Abstract

In cervical cancer (CC), cisplatin resistance greatly restricts the application in clinical. Here, we report that engineered exosomes-mediated transfer of hsa-miR-320a overcomes chemoresistance in cervical cancer cells via targeting Myeloid Cell Leukemia Sequence 1 (MCL1). In DDP resistant CC tissues, as well as cell lines, it was found that miR-320a expression is lower, engineered miR-320a exosomes were used to attenuate DDP resistance in Hela/DDP and Caski/DDP cells. Mechanistically, we find that MCL1, which is a target of miR-320a, overcomes DDP resistance in Hela/DDP cells and in mice. In conclusion, we report that the engineered miR-320a exosomes is proved to be effective and safe.

## Introduction

In women, CC is the fourth malignant cancer (Arbyn et al., 2020). The causes that associated with the development of CC include human papillomavirus (HPV) infection, reproductive factors, long-term oral contraceptives, smoking, obesity, genetic changes, epigenetic aberrations, and so on (Arora et al., 2019).

The CC patient survival rate is low because of its metastasis and recurrence, even there are progress in surgical and r chemoradiotherapy (Pfaendler et al., 2018). Nowadays, cisplatin (DDP)-based chemoradiotherapy is the main treatment. However, cisplatin resistance is a big challenge for the clinical application (Zhu et al., 2016). Overcoming cisplatin resistance could improve the efficacy of cisplatin-based therapy.

MiR-320a is related to chemoresistance and cisplatin resistance in lung adenocarcinoma (Lu et al., 2020) and laryngeal carcinoma (Yuan et al., 2018), doxorubicin resistance in osteosarcoma (Zhou et al., 2018), imatinib resistance in gastrointestinal stromal tumors (Gao et al., 2014), 5-FU resistance in human pancreatic cancer (Wang et al., 2016), and tamoxifen resistance in other type such as breast cancer (Lu et al., 2015). Whether miR-320a is involved in cisplatin resistance in cervical cancer is unknown.

Exosomes are nano-sized vesicles with a 30–150 nm diameter, and are enclosed by a lipid bilayer, nucleic acids or miRNAs (Dong et al., 2021). Exosomes can be transferred to influence the phenotype of recipient cells regulating and serving as a potential tool for therapy. Compared with liposome and nanoparticle, the exosomes are with high efficiency and biocompatibility.

It was reported that MCL1 is a key molecule in cisplatin resistance, such as. MCL1 in non-small cell lung cancer (NSCLC) (Ma et al., 2016; Lu et al., 2021) and hypopharyngeal squamous cell carcinoma (Liu X. et al., 2021). The miR-320a/MCL1 axis mediates the doxorubicin resistance of osteosarcoma (Zhou et al., 2018). Thus, we hypothesis that miR-320a/MCL1 may mediate DDP resistance in cervical cancer.

Here, we report that miR-320a expression is lower in DDP resistant CC, and engineered miR-320a exosomes can attenuate DDP resistance. The miR-320a molecule directly combines with and negatively regulates MCL1, resulting in alleviation of DDP resistance. The effectiveness and safety of engineered miR-320a exosomes in DDP resistant cervical cancer provides a new therapy strategy.

## Materials and Methods

### Cells and Animals

Hela and Caski cell lines were from Shanghai Culture Collection of Chinese Academy of Sciences. Hela and Caski cells were cultured in RPMI-1640 (Gibco, United States) medium. The medium contains 10% fetal bovine serum (FBS, Gibco, United States) with 100 U/ml penicillin/streptomycin (Gibco, United States) at 37°Cand 5% CO_2_ in a humidified incubator.

C57BL/6J female mice of 8–10 weeks were from Shanghai SLAC Laboratory Animal Co., Ltd. (Shanghai, China). Mice were maintained at 25 ± 1°C under light and dark cycles. Three mice were randomly selected for each experiment. All animal experiments were approved by the Animal Care Committee of Zhejiang University School of Medicine.

### qRT-PCR

The total RNA was isolated from cells using Trizol (Invitrogen) and quantified with Nanodrop 2000 (Thermo Fisher Scientific). qPCR was performed on ABI 7300 Thermocycler (Thermo Fisher Scientific) with SYBR Premix Ex Taq kit (Thermo Fisher Scientific). We use GAPDH and U6 as internal control. The relative expressions were calculated by the 2^−ΔΔCt^ method. The sequences employed are as follows: GAPDH forward (F): 5ʹ-AAC​GGA​TTT​GGT​CGT​ATT​GG-3ʹ and Reverse (R): 5ʹ-TTG​ATT​TTG​GAG​GGA​TCT​CG-3ʹ; MCL1 F: 5ʹ-CCA​GAG​GTG​AAC​CAC​AGC​G-3ʹ, and R: 5ʹ-AGC​CCC​TGT​CAA​TCC​TCC​T-3ʹ; U6 F: 5ʹ-CTC​GCT​TCG​GCA​GCA​CA-3ʹ, and R: 5ʹ-AAC​GCT​TCA​CGA​ATT​TGC​GT-3ʹ; miR-320a, 5′-AAA​AGC​UGG​GUU​GAG​AGG​GCG​A-3′.

### MTT Assay

The cells were plated in 96-well plates at a density of 5,000 cells/well, incubated overnight, and then treated. Cells were incubated with 20 μl MTT for 4 h at 37°C. The formazan crystals were dissolved in 150 μl DMSO, and the OD490 values were measured with a BioTek instrument (Winooski, Vermont, United States).

### Engineered miR-320a Exosome Preparation and Identification

A miR-320a lentiviral vector was from GenePharma, Shanghai. HEK293 cells were incubated with retroviral supernatant and selected by puromycin dihydrochloride (Thermo Fisher). The conditioned medium (CM) was harvested to isolate exosomes. Briefly, the CM was centrifuged at 300×g (15 min) and 2000×g (15 min) and filtered by a 0.22-μm filter (Merck Millipore, United States). Then, the solution was ultracentrifuged at 100,000×g (90 min) in an ultracentrifuge (Beckman, United States). The pellets were resuspended in PBS and ultracentrifuged at 100,000×g (90 min). In order to quantify the exosomes, Nanoparticle Tracking Analysis was used. Transmission electron microscopy (TEM) was used for the morphology of exosomes.

### Western Blotting

For protein extraction, cells were washed and suspended with RIPA buffer. The protein was obtained by centrifugation with 12,000 rpm for 30 min at 4°C. Protein was separated by 10% SDS-PAGE. The bands were transferred onto nitrocellulose membrane (PALL, United States), then blocked with 5% skim milk and incubated with primary conjugated antibodies (CD63: Proteintech, Cat No. 25682-1-AP; TSG101: Proteintech, Cat No. 67381-1-Ig; Bax: Proteintech, Cat No. 50599-2-Ig; Bcl-2: Proteintech, Cat No. 12789-1-AP; Cleaved Caspase3: CST, Cat No. 9661; Cleaved Caspase9: CST, Cat No. 7237; MCL1: Proteintech, Cat No. 16225-1-AP; β-actin: Proteintech, Cat no. 66009-1-Ig). Then the nitrocellulose membrane was incubated with the secondary conjugated antibody (HRP-conjugated Affinipure Goat Anti-Rabbit: Proteintech, Cat no. SA00001-2; HRP-conjugated Affinipure Goat Anti-Mouse IgG: Proteintech, Cat no. SA00001-1) for 4 h at room temperature and washed with TBST. The target protein bands were imaged by ECL (Advansta, United States), analyzed, and quantified by Bio-Rad ChemiDoc™ MP system (Bio-Rad, United States).

### CCK-8 Assay

For the proliferation assay, a CCK-8 kit (Beyotime Biotechnology Inc., China) was used. Cells were seeded into 96-well plates at a density of 2 × 10^3^ cells/well and incubated for 24, 48, 72 or 96 h at 37°C. Then, 20 µl of 10% CCK-8 reagent was added per well and incubated at 37°C for 2 h. The optical density was measured at 450 nm using an Enzyme Immunoassay Analyzer (Bio-Rad Laboratories, Inc.).

### Apoptosis Analysis

The flow cytometry was used for the apoptosis. The cell pellets from each group were harvested, collected, and dissolved in 500 μl binding buffer, incubated with 5 μl AnnexinⅤ-FITC and 5 μl PI in darkness for 30 min at RT and immediately subjected to flow cytometry analysis.

### Luciferase Reporter Assays

The 3′UTR of MCL1 containing miR-320a binding sites were synthesized by Genepharma (Shanghai, China). These fragments were linked to the luciferase reporter gene vector (Promega, Madison, WI, United States). The luciferase activity was measured using the Dual-Luciferase Reporter Assay System (Promega, United States).

### RIP Assay

The EZ-Magna RIP Kit (Sigma, St. Louis, MO, United States) was used for the RIP assay. In brief, the lysate of 1 × 10^7^ cells in RIP lysis buffer was incubated with Anti-IgG or Anti-Ago2-coated magnetic beads for 6 h. RNAs of MCL1 and miR-320a enriched on the beads was extracted and quantified by reverse-transcription quantitative polymerase chain reaction (qRT-PCR).

### TUNEL Assay

Fixed cells were used for TUNEL assay (Beyotime, Shanghai, China). Briefly, the cells were incubated with 50 μl of TUNEL reaction buffer for 50 min and diaminobenzidine (DAB) for 3 min, the results were photographed using an epifluorescence microscope at ×400 magnification (Nikon Eclipse 80i).

### Immunofluorescence Staining

For immunofluorescence Staining, the mainly procedure was described in previously report (Zhang et al., 2022). Briefly, the treated Hela/DDP and Caski/DDP cells were washed by PBS and fixed by ice chilled methanol. The exosome was labeled by PKH26 (Puzar Dominkus et al., 2018) using PKH26 Red Fluorescent Cell Linker Kits for General Cell Membrane Labeling (Sigma-Aldrich). DAPI (Sigma-Aldrich) stained cellular nucleus with following the instruction, slides were imaged by confocal laser scanning microscopy (Olympus).

### Hematoxylin-Eosin (HE) Staining and Immunohistochemistry (IHC) Assays

Dissected solid tumors were fixed with 10% formalin and imaged by a microscope at 400×. For IHC assays, the paraffin slices were dewaxed by xylene, rehydrated by gradient ethanol, immerged in 3% H_2_O_2_ for 30 min, then the primary antibody (PCNA: Proteintech, Cat No. 24036-1-AP) was incubated at 4°C overnight. Then, the slides were incubated with peroxidase-conjugated streptavidin at 37°C for 25 min. The slides were counterstained with hematoxylin, dehydrated by gradient ethanol and clarified by xylene, imaged by a microscope (Olympus, Japan) with 200×. The brown staining indicates the positive reactions.

### Statistical Analysis

Overall survival analysis was performed using the Kaplan-Meier method. All data are presented as mean ± SD. Data were analyzed by ANOVA, followed by Turkey post hoc test. A *p* value below 0.05 was considered that there were statistically significant differences. *, *p* < 0.05; **, *p* < 0.01; ***, *p* < 0.001.

## Results

### MiR-320a Expression is Lower in DDP Resistant CC Cells

**FIGURE 1:**
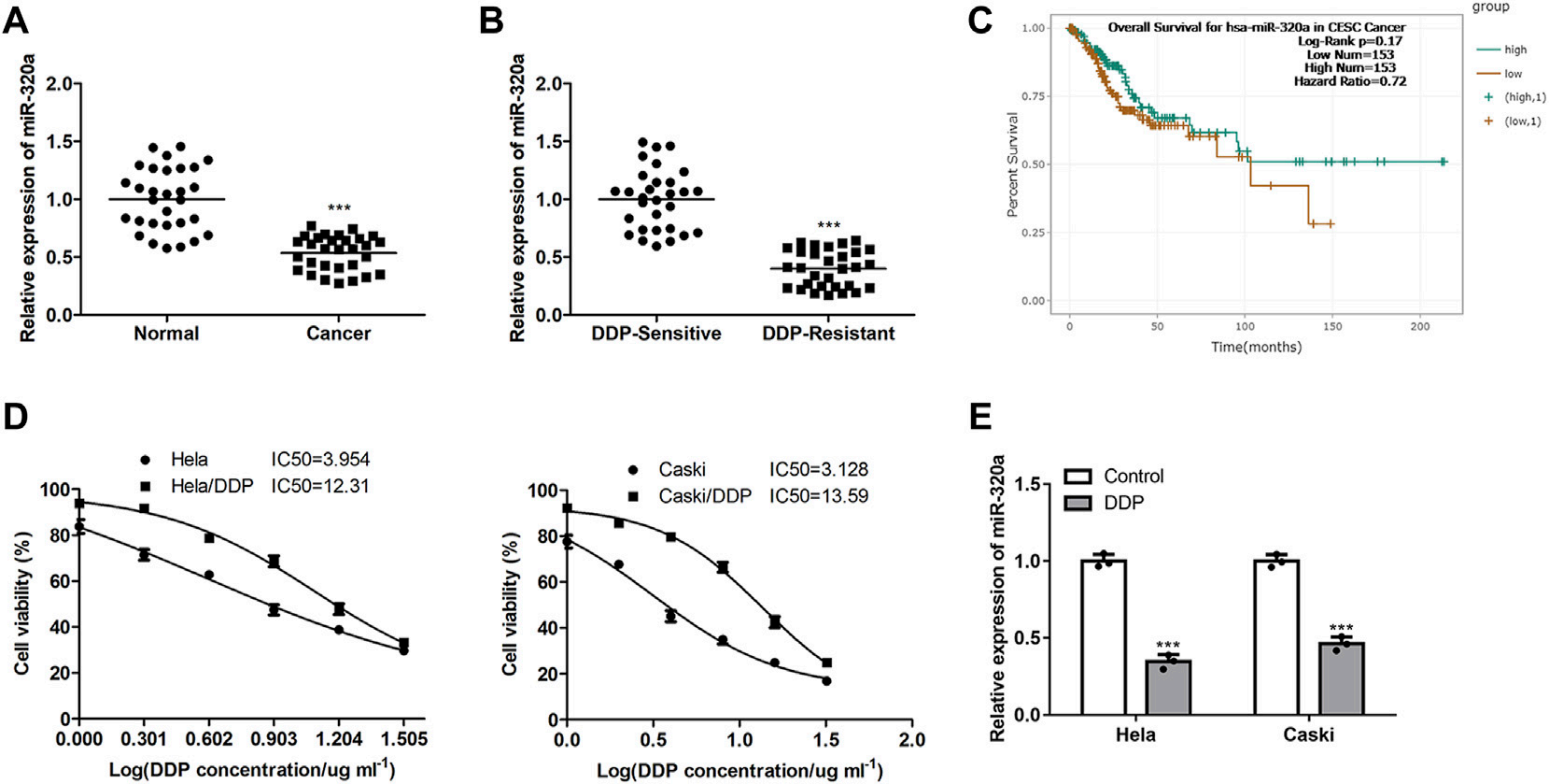
The miR-320a expression levels are inhibited in DDP resistant CC cells. **(A)** The miR-320a expressions in para-cancer and CC tissues were determined by qRT-PCR analysis. **(B)** The miR-320a expressions in DDP sensitive clinical CC tissues and DDP resistant clinical CC tissues were determined by qRT-PCR analysis. **(C)** The overall survival of miR-320a high- and low expression patients analyzed by the Kaplan-Meier method. **(D)** IC50 values of DDP resistant CC cell line Hela/DDP and Caski/DDP were verified by MTT assay. **(E)** The miR-320a expressions of DDP sensitive or resistant CC cell lines Hela and Caski were measured by qRT-PCR. ***, *p* < 0.001 vs. Control group.

Firstly, we found that the miR-320a expression were decreased in CC tissues (Figure 1A), and further decreased in DDP resistant CC tissues (Figure 1B). However, there were no significant differences in overall survival of patients between miR-320a high- and low-expression (Figure 1C). Next, after the DDP resistances of CC cell line Hela/DDP and Caski/DDP being verified (Figure 1D), miR-320a expression was also inhibited in these DDP resistant CC cell lines (Figure 1E). These data indicate MiR-320a expression is downregulated in DDP resistant CC cells.

### Engineered miR-320a Exosomes can be Effectively Up-Taken by Hela/DDP and Caski/DDP Cells

**FIGURE 2:**
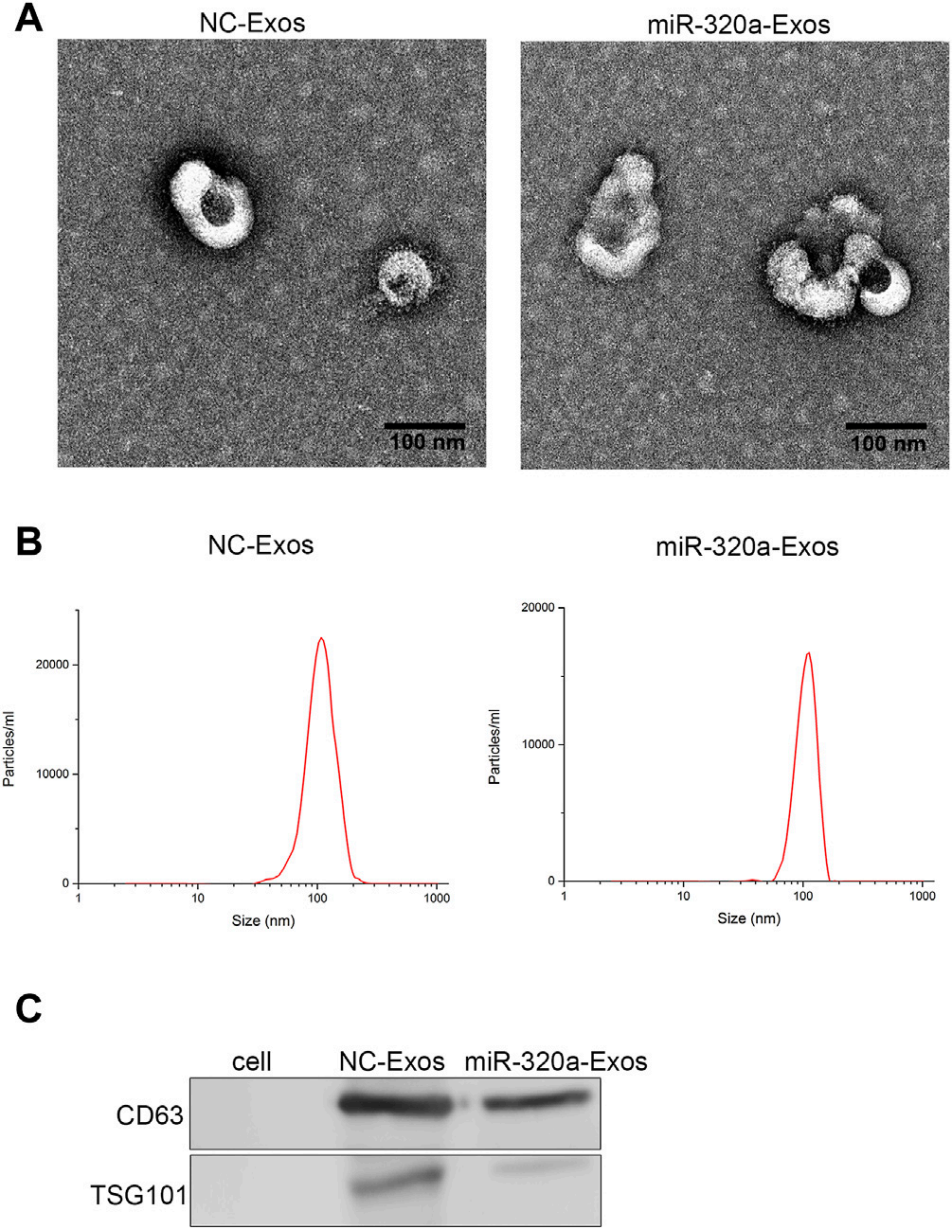
The characterization of engineered miR-320a exosomes. The morphology and diameter distribution of non-engineered HEK293 exosomes and engineered miR-320a exosomes were detected by TEM **(A)** and DLS **(B)**. **(C)** The exosomal specific markers CD63 and TSG101 were detected by Western blotting.

**FIGURE 3:**
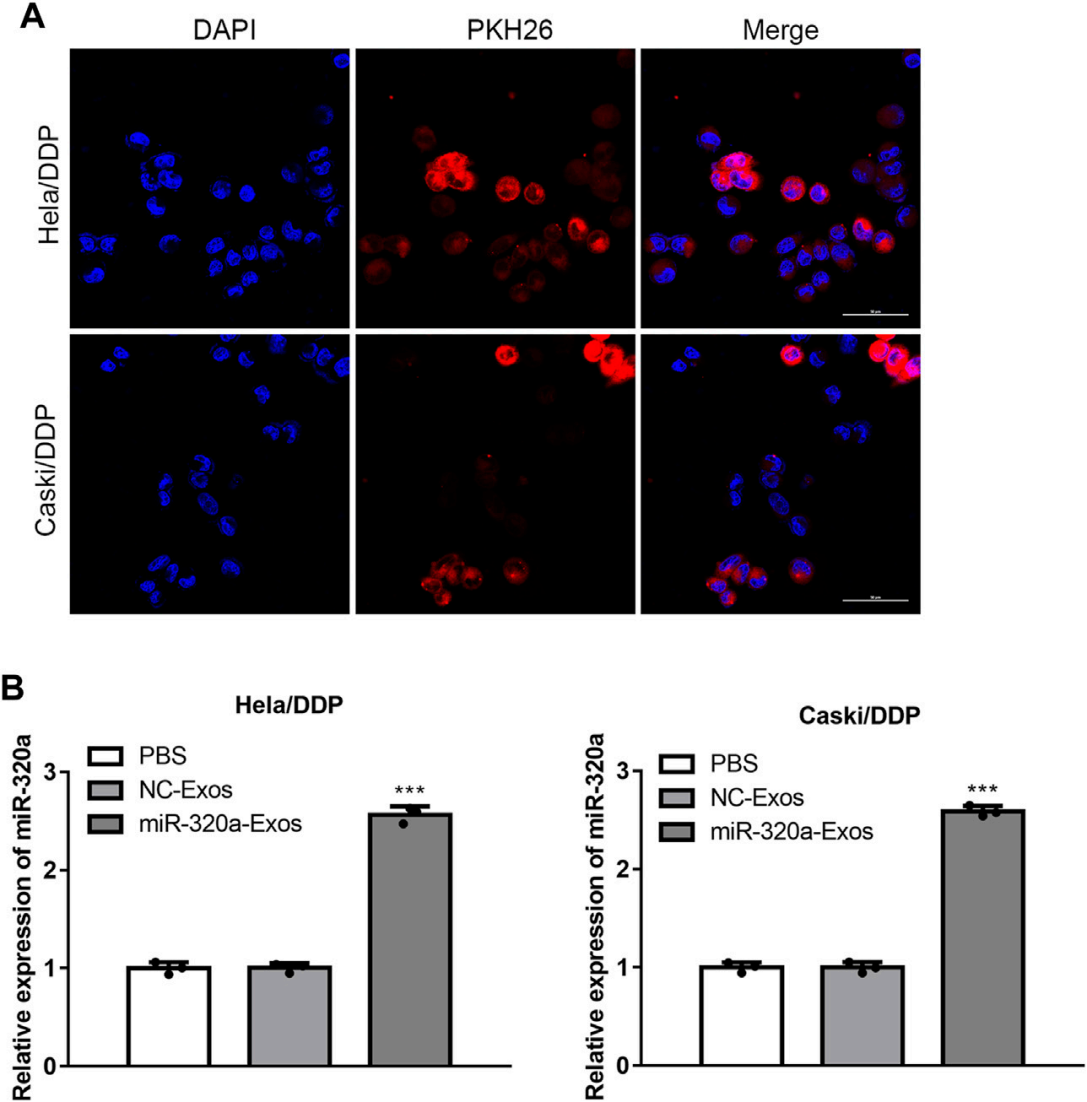
Engineered miR-320a exosomes can be effectively up-taken by Hela/DDP and Caski/DDP cells. **(A)** The uptake of engineered miR-320a exosomes by Hela/DDP and Caski/DDP was confirmed by laser confocal microscopy. **(B)** The miR-320a expression in Hela/DDP and Caski/DDP cells after internalizing the engineered miR-320a exosomes was determined by qRT-PCR. ***, *p* < 0.001 vs. PBS group.

MiR-320a overexpressing cells were constructed, And the exosomes were obtained. According to transmission electron microscopy (TEM) (Figure 2A) and dynamic light scattering (DLS) (Figure 2B), it was found that the engineered miR-320a exosomes were spherical microvesicles ranged from 40 to 130 nm. Western blotting for exosomal characteristic markers (CD63 and TSG101) further confirmed the specific molecule of the exosomes (Figure 2C). Moreover, these data indicate that there are no differences in morphology and specific markers between the non-engineered HEK293 exosomes and engineered miR-320a exosomes. Next, we observed that the engineered miR-320a exosomes could be phagocytized into both Hela/DDP and Caski/DDP cells via laser confocal microscopy (Figure 3A). The qRT-PCR analysis for miR-320a showed that miR-320a in Hela/DDP and Caski/DDP cells were promoted by engineered cell exosomes (Figure 3B). These results show that the engineered miR-320a exosomes are effectively up-taken by Hela/DDP and Caski/DD cells.

### Engineered miR-320a Exosomes Attenuate DDP Resistance in Hela/DDP and Caski/DDP Cells

**FIGURE 4:**
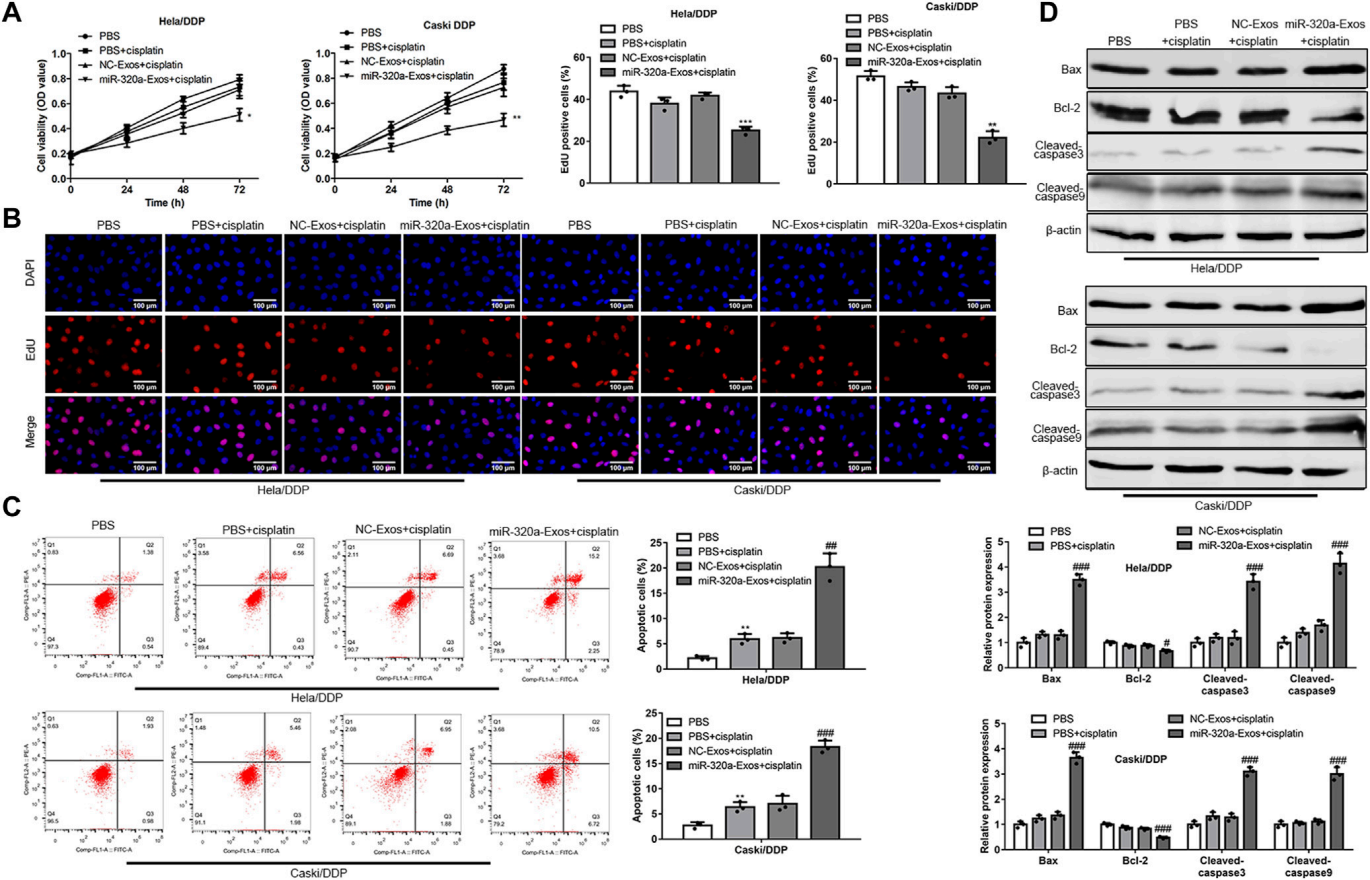
Engineered miR-320a exosomes attenuates DDP resistance in Hela/DDP and Caski/DDP cells. **(A)** The cell viability, proliferation, apoptosis rate, and apoptosis associated proteins of DDP induced Hela/DDP and Caski/DDP cells affected by engineered miR-320a exosomes were measured by CCK-8 assay **(A)**, 5-Ethynyl-2′-deoxyuridine (EdU) assay **(B)**, annexin V-FITC/PI apoptotic kit **(C)**, and Western blot **(D)**, respectively. *, *p* < 0.05; **, *p* < 0.01; ***, *p* < 0.001 vs. PBS group; #, *p* < 0.05; ##, *p* < 0.01; ###, *p* < 0.001 vs. PBS + cisplatin group.

To investigate whether the engineered miR-320a exosomes, which were up-taken by Hela/DDP and Caski/DDP cells, affected the cell sensitivity to DDP, the Hela/DDP and Caski/DDP cells were treated with exosomes from non-engineered HEK293 and engineered miR-320a exosomes, and stimulated with DDP (1ug/ml) at 24, 48, 72 h. The results suggested that, compared with non-engineered HEK293 exosomes, engineered miR-320a exosomes increased the inhibitory function of DDP on Hela/DDP and Caski/DDP cell viability (Figure 4A) and proliferation (Figure 4B). Additionally, engineered miR-320a exosomes enhanced the DDP induced apoptosis rates of Hela/DDP and Caski/DDP cells (Figure 4C). Consistent with the apoptosis rates, the levels of pro-apoptotic protein markers, Bax/Bcl-2 ratio, cleaved-caspase3, and cleaved-caspase9, were increased in the DDP stimulated Hela/DDP and Caski/DDP cells by engineered miR-320a exosomes (Figure 4D). These data indicate that engineered miR-320a exosomes overcome DDP resistant CC cell line Hela/DDP and Caski/DDP cells.

### MCL1 is a Target of miR-320a

**FIGURE 5:**
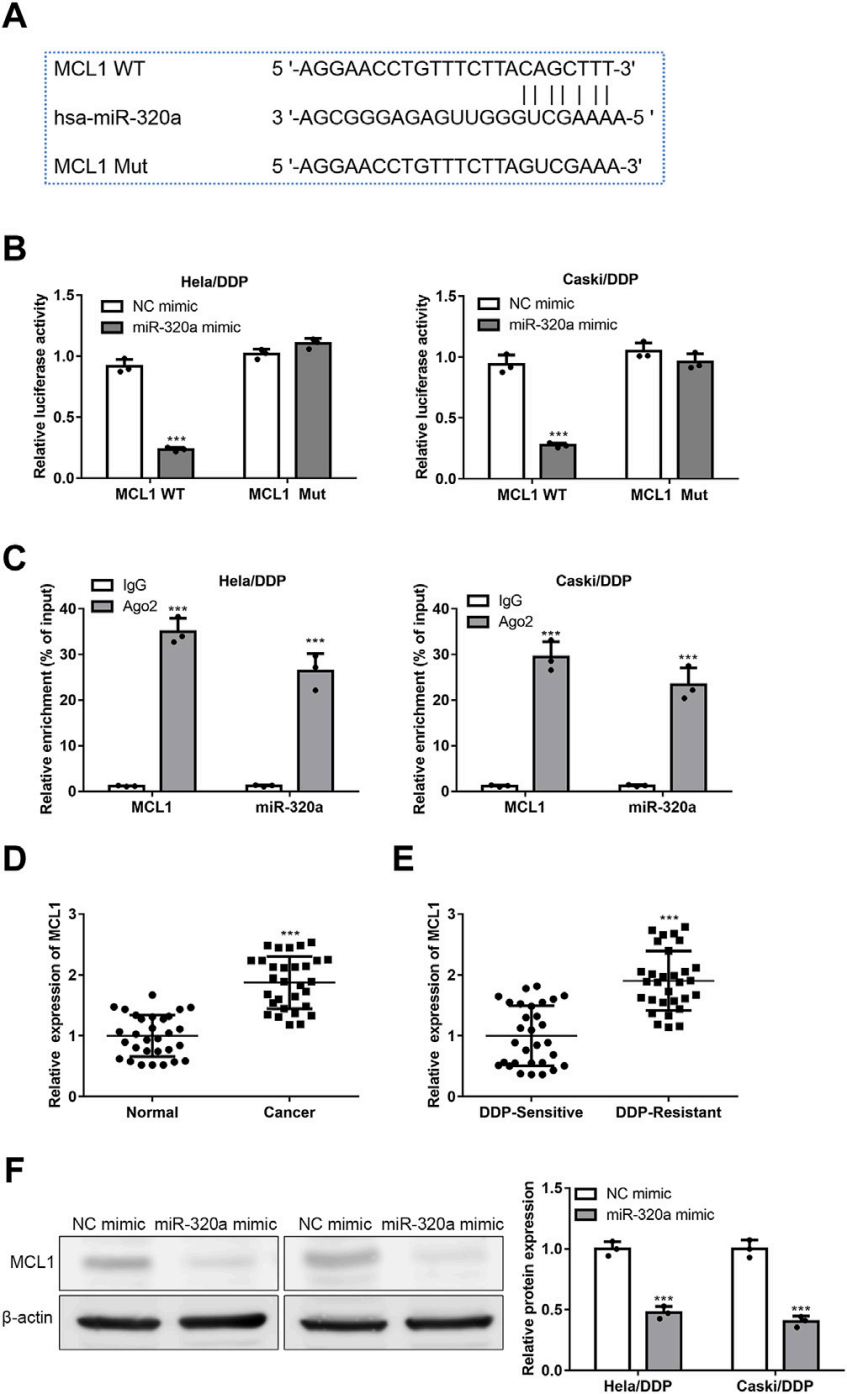
MCL1 is a target of miR-320a. **(A)** The complementary sequences of 3′-UTR of MCL1 mRNA and miR-320a were analyzed by StarBase website. **(B)** The level of MCL1 expression regulated by miR-320a is verified by the luciferase reporter assay. **(C)** The combination of MCL1 mRNA and miR-320a is confirmed by RIP assay. **(D)** The mRNA levels of MCL1 in CC tissues were detected by qRT-PCR. **(E)** The mRNA levels of MCL1 in DDP resistant CC tissues were detected by qRT-PCR. **(F)** The MCL1 proteins regulated by miR-320a were confirmed by Western blot. ***, *p* < 0.001 vs. Control group.

Among the potential targets of miR-320a, MCL1 has been reported as a target of miR-320a (PMID: 30119255). MCL1, as an anti-apoptotic protein, is a therapeutic target of CC (PMID: 32715755). Thus, our study focused on whether MCL1 is regulated by miR-320a in Hela/DDP and Caski/DDP cells. The bioinformatics results showed that the 3′-UTR of MCL1 mRNA displayed complementary sequences for miR-320a (Figure 5A). Then, dual-luciferase reporter assay was carried out, the results indicated the decreased of MCL1-WT luciferase activity in Hela/DDP and Caski/DDP cells in the addition of miR-320a, relative to MCL1-MUT (Figure 5B). And the results of RIP assay suggested that MCL1 mRNA was directly combined with miR-320a (Figure 5C). Next, we validated that the levels of MCL1 mRNA were increased in CC (Figure 5D) and DDP resistant CC (Figure 5E) in clinical samples. And MCL1 protein expression was proved to be downregulated by miR-320a in Hela/DDP and Caski/DDP cells (Figure 5F). These data indicate that MCL1 directly combines and negatively regulated by miR-320a.

### MiR-320a Overcomes DDP Resistance in Hela/DDP Cells by Regulating MCL1

**FIGURE 6:**
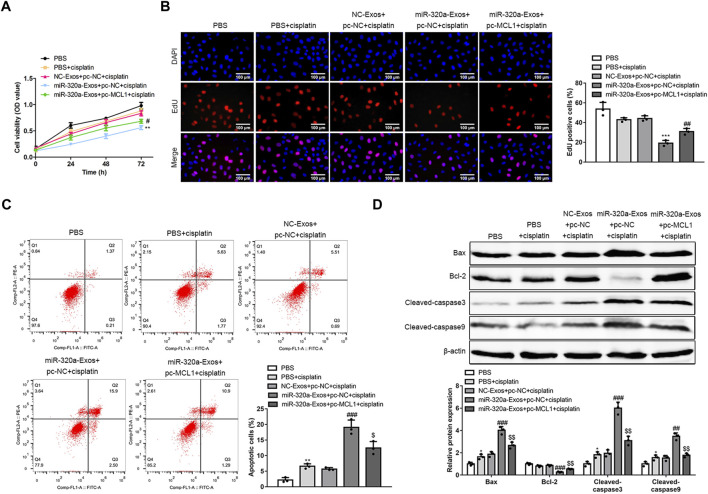
MiR-320a overcomes DDP resistance in Hela/DDP cells by regulating MCL1. **(A)** The cell viability, proliferation, apoptosis rate, and apoptosis associated proteins of DDP induced Hela/DDP and Caski/DDP cells affected by engineered miR-320a exosomes and MCL1 overexpression were measured by CCK-8 assay **(A)**, 5-Ethynyl-2′-deoxyuridine (EdU) assay **(B)**, annexin V-FITC/PI apoptotic assay **(C)**, and Western blot **(D)**, respectively. *, *p* < 0.05; **, *p* < 0.01; ***, *p* < 0.001 vs. PBS group; #, *p* < 0.05; ##, *p* < 0.01; ###, *p* < 0.001 vs. NC-Exo + pc-NC + cisplatin group; $, *p* < 0.05; $$, *p* < 0.01 vs. miR-320a-Exo + pc-NC + cisplatin group.

To further evaluate whether miR-320a regulate DDP resistance in Hela/DDP cells *via* MCL1, MCL1 was overexpressed in Hela/DDP cells. The data showed that, MCL1 overexpression abolished the suppression of cell viability (Figure 6A) and proliferation (Figure 6B) by engineered miR-320a exosomes in Hela/DDP cells. Additionally, MCL1 overexpression canceled the enhanced apoptotic rates (Figure 6C) and upregulated pro-apoptotic protein markers (Bax/Bcl-2 ratio, cleaved-caspase3, and cleaved-caspase9) (Figure 6D) induced by engineered miR-320a exosomes in Hela/DDP cells. It is evidenced by our data that miR-320a overcomes DDP resistance in Hela/DDP cells by regulating MCL1.

### Engineered miR-320a Exosomes Ameliorate DDP Resistance *In Vivo*

**FIGURE 7:**
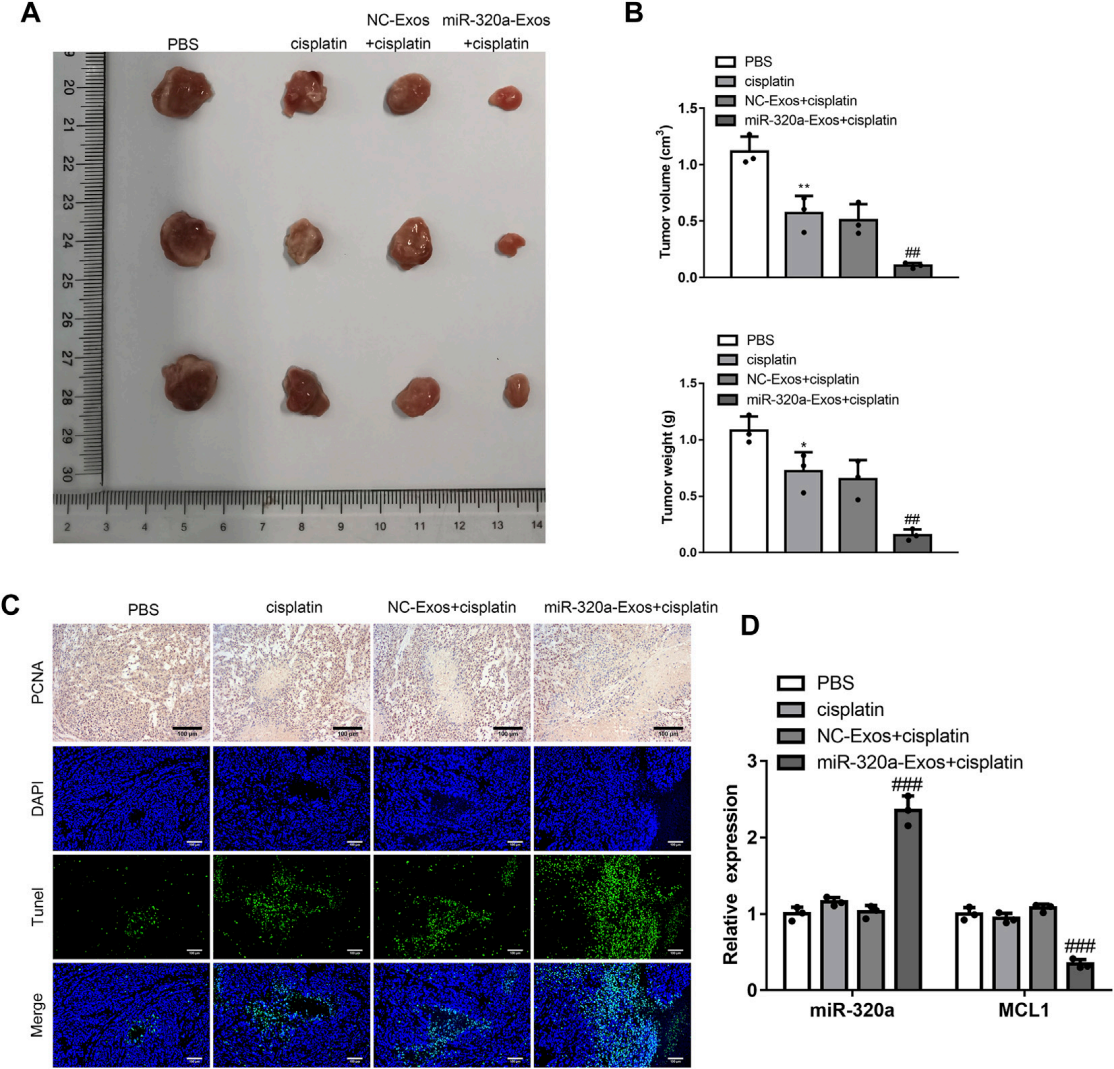
Engineered miR-320a exosomes ameliorate DDP resistance *in vivo*. **(A)** The tumor images, and. **(B)** The tumor volumes and weights of the mice with subcutaneous tumor formation of Hela/DDP cells affected by tail vein injection of engineered miR-320a exosomes. **(C)** The apoptosis and proliferation of tumor tissues detected by Tunel assay and immunohistochemistry. **(D)** MCL1 mRNA expression regulated by engineered miR-320a exosomes injection is evaluated by qRT-PCR. *, *p* < 0.05; **, *p* < 0.01 vs. PBS group; ##, *p* < 0.01; ###, *p* < 0.001 vs. NC-Exos + cisplatin group.

To evaluate the function of engineered miR-320a exosomes on DDP resistance *in vivo*, engineered miR-320a exosomes were isolated and injected into the tail vein of the mice with subcutaneous Hela/DDP cells tumor. Th volumes and weights of tumor were inhibited by engineered miR-320a exosomes injection (Figures 7A,B). The Tunel assay showed that engineered miR-320a exosomes enhanced the apoptosis of tumor cells in the mice with subcutaneous tumor formation of Hela/DDP cells (Figure 7C), which is consistent with the inhibited proliferation *in vitro* (Figure 7C). Then, the expression of MCL1 mRNA was suppressed by the injection of engineered miR-320a exosomes (Figure 7D). These data are in consistent with *in vitro*, suggesting that engineered miR-320a exosomes overcome DDP resistance in CC.

### The Safety of Engineered miR-320a Exosomes Injection *In Vivo*

**FIGURE 8:**
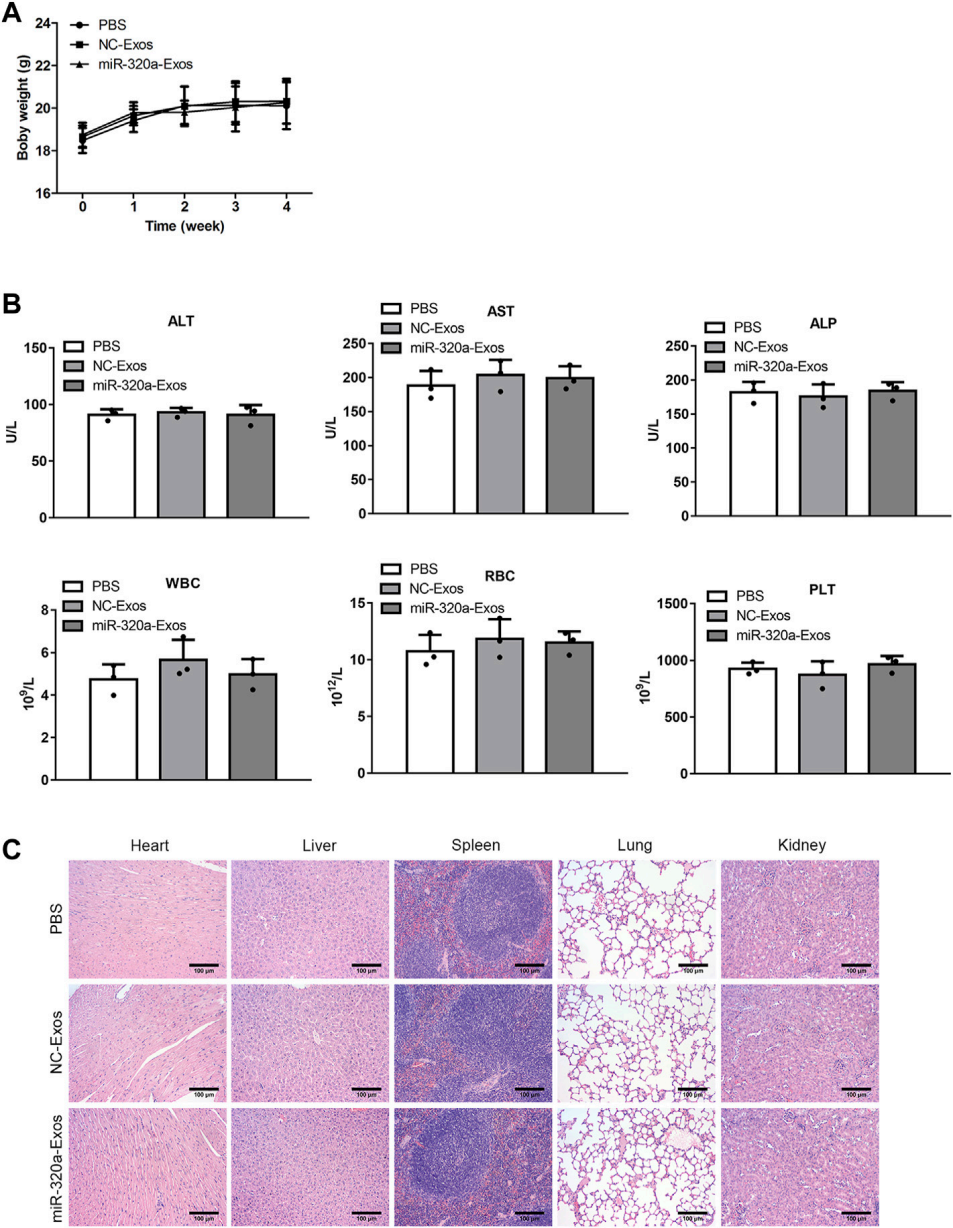
The safety of engineered miR-320a exosomes injection *in vivo*. **(A)** The body weights between the non-engineered HEK293 exosomes injected mice and the engineered miR-320a exosomes injected mice. **(B)** The differences of ALT, AST, ALP, WBC, RBC, and PLT in the serum of the engineered miR-320a exosomes injected mice compared with the non-engineered HEK293 exosomes injected mice. **(C)** The histopathological staining of heart, liver, spleen, lung, and kidney of the engineered miR-320a exosomes injected mice compared with the non-engineered HEK293 exosomes injected mice.

We evaluated the safety of engineered miR-320a exosomes which were injected into the tail vein. No significant difference was found in the body weights between the non-engineered HEK293 exosomes injected mice and the engineered miR-320a exosomes injected mice (Figure 8A). Furthermore, ALT, AST, ALP, WBC, RBC, and PLT in the serum were not different in the engineered miR-320a exosomes injected mice compared with the non-engineered HEK293 exosomes injected mice (Figure 8B). And the histopathological staining of heart, liver, spleen, lung, and kidney did not show observable change in the engineered miR-320a exosomes injected mice compared with the non-engineered HEK293 exosomes injected mice (Figure 8C). These data indicate that tail vein injection of engineered miR-320a exosomes is safe.

## Discussion

Although major progress has been made in improving the effectiveness of DDP for CC chemotherapy, DDP resistance remains a serious problem, which is urgent to be solved. Here, we confirm the effectiveness and safety of engineered miR-320a exosomes in DDP resistant cervical cancer. And the bioinformatics analysis and the dual-luciferase reporter assay confirms that miR-320a directly binds to the 3′-UTR of MCL1 mRNA and negatively regulates its function, improving the DDP chemoresistance.

MiR-320a has been considered as an inhibitor of carcinoma. A recent study showed that low level of miR-320a promoted glioma proliferation through FOXM1 expression (Qi et al., 2021). Additionally, it was reported that miR-320a, the expression of which is modulated by demethylation, ameliorated DDP resistance in lung adenocarcinoma (Lu et al., 2020). Moreover, as a downstream target of long non-coding RNA AFAP1-AS1, the low expression of miR-320a promoted the stemness and DDP chemoresistance of laryngeal carcinoma cells by negative regulation of RBPJ mRNA and protein (Yuan et al., 2018). Consistent with these results, here, we verified the effectiveness of miR-320a in DDP resistant cervical cancer.

Exosomes are nano-sized vesicles with the diameter of 30–150 nm, they contain various biomolecules, and can be secreted by almost all mammalian cell types. Based on the structure, engineered exosomes have become a possibility in the treatment of numerous diseases. A recent study indicated that miR-317b-5b-loaded engineered exosomes can transfer itself both *in vitro* and *in vivo* for the anti-tumor functions (Xue et al., 2021). In addition, engineered miR-181b exosomes regulate macrophage polarization, thus to improve osteointegration (Liu W. et al., 2021). In this study, engineered miR-320a exosomes were used to alleviate DDP resistance in CC.

MCL1, a pro-survival and pro-proliferative factor, plays a critical role in many tumor types. The deubiquitination and stabilisation of MCL1 was considered to be required for the proliferation of CC cells (Morgan et al., 2021). What’s more, MCL1 siRNA-silencing demonstrated high efficacy on CC cell lines (Lechanteur et al., 2017). All these data support that MCL1 is a therapeutic target for CC. In contrast, we confirmed that MCL1 functioned as the target of miR-320a for ameliorating DDP resistance in CC, which was consistent with the previous study that miR-320a/MCL1 axis mediated the doxorubicin resistance of osteosarcoma (Zhou et al., 2018).

Besides the effect we mentioned above, miR-320a has a great number of functions in numerous aspects. For example, by inhibiting MafF, the pancreatic beta cells loss its function in diabetes (Du et al., 2021); it also regulates endoplasmic reticulum stress as well as autophagy in diffuse large B-cell lymphoma (Cui et al., 2021); and human glucagon expression (Jo et al., 2021). Although the safety of engineered miR-320a exosomes was assessed by measuring body weights, ALT, AST, ALP, WBC, RBC, and PLT in the serum, and the histopathological staining, more comprehensive evaluation is required to investigate the impact of miR-320a.

In conclusion, we confirmed that miR-320a is downregulated in DDP resistant CC and engineered miR-320a exosomes can attenuate DDP resistance by inhibiting MCL1.

## Data Availability

The original contributions presented in the study are included in the article/Supplementary Material, further inquiries can be directed to the corresponding authors.
